# Oral vancomycin in the treatment of primary sclerosing cholangitis-associated pouchitis

**DOI:** 10.1093/gastro/goab004

**Published:** 2021-02-05

**Authors:** Bo Shen

**Affiliations:** Center for Inflammatory Bowel Disease, Columbia University Irving Medical Center, New York Presbyterian Hospital, New York, NY, USA

**Keywords:** pouchitis, primary sclerosing cholangitis, vancomycin

## Introduction

Pouchitis is a common complication in those with restorative proctocolectomy with ileal pouch–anal anastomosis (IPAA). Some patients may develop chronic antibiotic-refractory pouchitis and its management can be challenging. Primary sclerosing cholangitis (PSC) has consistently been reported to be a risk factor for chronic pouchitis. Patients with PSC-associated pouchitis and enteritis may represent a unique phenotype [1] that usually does not respond to conventional antibiotics, such as metronidazole and ciprofloxacin. PSC-associated pouchitis has been treated with budesonide [2]. Vancomycin has been explored in the treatment of PSC [3], while the agent is routinely used for the treatment of *Clostridium difficile*-associated pouchitis [4].

## Case presentation

A 48-year-old male with a history of ulcerative colitis (UC) and PSC presented with frequent and loose stools ever since total proctocolectomy with IPAA 10 years prior. Other symptoms include postprandial pelvic and abdominal pain, nausea, urgency, incontinence, nocturnal seepage, and weight loss of 15 pounds over the past 2 months. He was an active smoker. The patient washed had been diagnosed with having pouchitis and enteritis on pouchoscopy and histology. His past treatment regimen included metronidazole, ciprofloxacin, oral and topical mesalamines, hydrocortisone enemas, and oral prednisone with minimal relief of symptoms. He was on long-term oral budesonide with recurrent symptoms while tapering off the corticosteroid. He underwent multiple pouchoscopies that showed no improvement in mucosal inflammation in the pouch body and afferent limb.

At the time of his initial presentation at our Pouch Center, he reported urgency, bowel frequency 12–15 times per day, loose-to-watery stools, occasional bleeding, incontinence, and nocturnal seepage. The Pouchitis Disease Activity Index (PDAI) symptom subscore was 5. Laboratory evaluation showed white blood cell count 7,600/μL, hemoglobulin 11.3 g/dL, platelet count 110,000/μL, creatinine 0.8 mg/dL, albumin 3.4 g/dL, alkaline phosphatase 320 international unit/L, total bilirubin 0.8 mg/dL, aspartate transaminase 23 international unit/L, and alanine transaminase 14 international unit/L.

**Figure 1.**
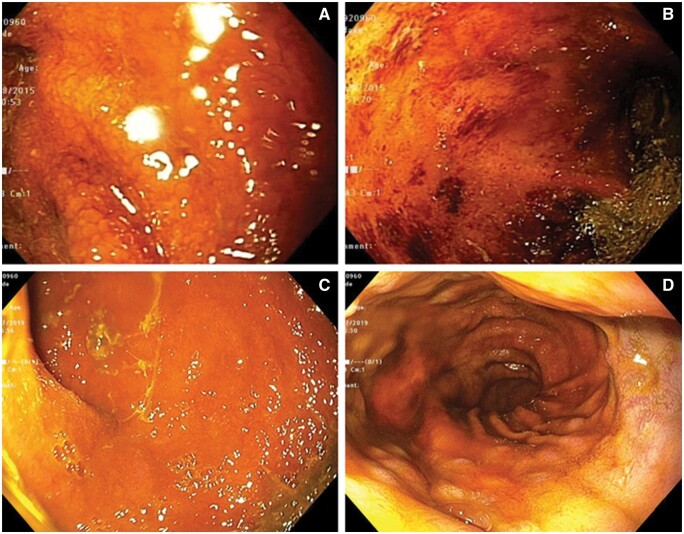
Pouchitis and enteritis before and after therapy with oral vancomycin. Diffuse hemorrhagic mucosal inflammation of the afferent limb (A) and pouch body (B) before the therapy. Resolution of inflammation of the afferent limb (C) and pouch body (D) after the therapy.

Pouchoscopy showed diffuse inflammation of both the pouch body and prepouch afferent limb with granularity, edema, loss of vascularity, and spontaneous bleeding. The PDAI endoscopy subscores of the pouch body and afferent limb were 4 and 2, respectively. The pouch inlet was widely patent. He was started on oral vancomycin 250 mg four times a day for the treatment of pouchitis, enteritis, and PSC. The patient responded with the PDAI symptom subscore reduced to 1. Fatigue also improved. Repeat pouchoscopy after 3 months showed mucosal healing with no active disease in the pouch body and afferent limb with PDAI endoscopy subscores of 0 and 1, respectively. The liver-function test showed that alkaline phosphatase was 120 international unit/L. He has been followed up for 4 years until now and a year later repeat pouchoscopy showed pouchitis and enteritis in remission (Figure 1). Alkaline phosphatase was 127 international unit/L.

## Discussion

Ilealpouch patients with concurrent PSC often have diffusepouch as well as afferentlimb inflammation. Similar to PSC-UC [5], PSC-pouchitis/enteritis may present a distinct phenotype of the immune-mediated disorder. Concurrent chronic pouchitis and PSC have been treated with vedolizumab [6] and oral budesonide [3], with symptomatic and endoscopic improvement in pouchitis in the absence of response in liver-function tests.

Oral vancomycin has been investigated for the treatment of PSC and PSC-UC [7]. The therapeutic effect was described in the control of disease activity of UC, but not PSC. Oral or intravenous vancomycin has been routinely used as one of the first-line therapies for *Clostridium difficile*-associated pouchitis [4]. Here, we reported that the patients with concurrent PSC-pouchitis/enteritis had a favorable response to oral vancomycin.

## Funding

None.
